# A microprotein encoded by *FERMT3* modulates endothelial cell protein catabolism and induces cell cycle arrest and senescence

**DOI:** 10.1186/s12964-026-03019-3

**Published:** 2026-06-25

**Authors:** Manav Raheja, Beyza Güven, Witold Szymanski, Stefan Günther, Carsten Kuenne, Vladislav Rakultsev, Marta Segarra, Süleyman Bozkurt, Christian Münch, Manuel Kaulich, Johannes Graumann, Ingrid Fleming, Mauro Siragusa

**Affiliations:** 1 Goethe University, Institute for Vascular Signalling, Centre for Molecular Medicine, Frankfurt am Main, Germany; 2 Philipps-Universität Marburg, Institute of Translational Proteomics & Core Facility Translational Proteomics, Biochemical/Pharmacological Centre, Marburg, Germany; 3 Max Planck Institute for Heart and Lung Research, Bioinformatics and Deep Sequencing Platform, Bad Nauheim, Germany; 4 Goethe University, Institute of Cell Biology and Neuroscience, Buchmann Institute for Molecular Life Sciences, Frankfurt am Main, Germany; 5 Goethe University, Institute of Molecular Systems Medicine, Faculty of Medicine, Frankfurt am Main, Germany; 6 Goethe University, Institute of Biochemistry II, Faculty of Medicine, Frankfurt am Main, Germany; 7 Frankfurt Cancer Institute, Frankfurt am Main, Germany; 8 German Centre for Cardiovascular Research (DZHK), Partner Site RheinMain, Frankfurt am Main, Germany; 9 CardioPulmonary Institute, Frankfurt am Main, Germany

**Keywords:** smORF, Microprotein, Endothelial cell, Protein ubiquitination, Proteasome, Senescence

## Abstract

**Background:**

Endothelial cells express numerous microproteins (miPs) encoded by small open reading frames (smORFs), yet the biological function of most remains unknown. This study set out to characterize a novel 69 amino acid miP encoded within the FERM domain containing kindlin-3 transcript (miP-FERMT3), which is upregulated under inflammatory conditions.

**Methods:**

Confocal microscopy was used to determine miP-FERMT3 localization, and its interaction partners were determined by mass spectrometry and immunoblotting. RNA sequencing and quantitative mass spectrometry were performed to assess transcriptional and proteomic alterations. Cell proliferation and cell cycle progression were examined by live cell imaging, EdU incorporation and flow cytometry, while senescence was determined by β-galactosidase staining, live cell imaging and RT-qPCR-based analysis of telomere length.

**Results:**

In endothelial cells, miP-FERMT3 localized mainly to centriole subdistal appendages, where it colocalized with ninein and CEP170 and induced centrosome amplification. The expression of miP-FERMT3 caused cell cycle arrest and DNA damage, evidenced by γ-H2AX foci and nuclear p53 accumulation. Consistent with this, miP-FERMT3-expressing endothelial cells exhibited downregulation of genes required for cell-cycle progression and upregulation of genes involved in cell cycle inhibition and senescence. However, canonical p53 target genes were not induced and cell cycle arrest occurred independently of p53. Mechanistically, miP-FERMT3 interacted with proteins involved in ubiquitin/proteasome-dependent protein catabolism, including PSMD9, CUL2 and TRIM8, and its expression increased protein ubiquitination, centrosomal neddylation and proteasomal activity. Notably, enhanced proteasomal turnover of p21 in miP-FERMT3-expressing endothelial cells resulted in replication stress, as evidenced by increased CHK1 phosphorylation. These alterations culminated in rapid induction of cellular senescence, characterized by enlarged cell size, β-galactosidase activity, telomere shortening and a paracrine pro-inflammatory activation of naïve endothelial cells. Analyses of independent murine and human transcriptomic and proteomic aging datasets further revealed that FERMT3 expression and protein abundance increase with age.

**Conclusions:**

miP-FERMT3 is a novel regulator of protein catabolism that promotes p21 degradation, replication stress and p53-independent cell cycle arrest and senescence in endothelial cells. Given the aging-associated upregulation of *FERMT3* in mouse and human endothelial cells, increased miP-FERMT3 expression may contribute to the onset of vascular senescence as a hallmark of aging.

**Supplementary Information:**

The online version contains supplementary material available at 10.1186/s12964-026-03019-3.

## Abstract (plain language summary)

A large number of previously uncharacterized small proteins, known as microproteins, have recently been identified in endothelial cells, the cells that line blood vessels. In this study, we investigated a microprotein that is more abundant in endothelial cells under inflammatory conditions linked to cardiovascular disease. We named this microprotein miP-FERMT3 because it is produced by non-canonical translation of the *FERMT3* transcript. In endothelial cells, miP-FERMT3 localized mainly to the centrosome, a structure involved in organizing cell division. Increased levels of miP-FERMT3 altered the centrosome, caused DNA damage and prevented endothelial cells from progressing normally through the cell cycle. As a result, the cells stopped proliferating and developed features consistent with cellular senescence, a state in which cells remain alive but no longer divide and can promote inflammation. We also found that miP-FERMT3 interacted with the cellular machinery responsible for protein breakdown and increased its activity. This led to increased loss of p21 and other proteins needed for normal control of the cell-cycle, providing a possible explanation for the growth arrest and cellular senescence in miP-FERMT3-expressing endothelial cells. Finally, expression of the *FERMT3* transcript was higher in endothelial cells from old mice and humans. As blood vessels are among the first tissues to show signs of aging, miP-FERMT3 may represent a link between inflammation and vascular aging.

**Supplementary Information:**

The online version contains supplementary material available at 10.1186/s12964-026-03019-3.

## Introduction

The genome contains thousands of open reading frames (ORFs) i.e., sequences between in-frame start and stop codons. ORFs with a maximum length of 300 nucleotides are referred to as small open reading frames (smORFs) and are located within transcripts previously annotated as both protein-coding and non-coding [1–4]. While some smORFs are ubiquitously expressed, others demonstrate high cell and tissue specificity [5]. Historically, the latter were overlooked and presumed to lack biological relevance but many of these elements are now recognized as being functionally important. Some well-characterized examples reside in the 5′ untranslated regions (UTRs) of mRNAs where they influence the translation efficiency of the downstream main coding sequence [6]. In addition, many smORFs encode short peptides of 100 or fewer amino acids, commonly referred to as microproteins (miPs) that can be detected by advanced mass spectrometry-based proteomic approaches [2, 3, 7]. Despite their relatively poor conservation across species [4–6, 8], miPs have been shown to contribute to a range of fundamental processes including DNA repair [2], calcium signalling [9–11], stress responses [12] and cell cycle control [13].

Endothelial cells express a large repertoire of previously unannotated smORF-encoded miPs [5, 7] that vary across organs and inflammatory status [7]. Despite the limited sequence conservation between human and murine smORFs, we identified several smORFs whose expression was altered in interleukin (IL)−1β-treated human endothelial cells and in endothelial cells from a mouse model of endothelial dysfunction and accelerated atherogenesis. One of these smORFs is located within the coding sequence of the *FERM Domain Containing Kindlin 3* (*FERMT3*) transcript (ENST00000345728.10) but is translated in an alternative reading frame than the canonical ORF. Translation of this internal ORF is increased under inflammatory conditions both in vitro and in vivo, producing a 69 amino acid miP, hereafter referred to as miP-FERMT3. As this represents a previously uncharacterized miP, we investigated its function in human endothelial cells.

## Results

### miP-FERMT3 localizes to cytoplasm and centriole subdistal appendages

**Fig. 1:**
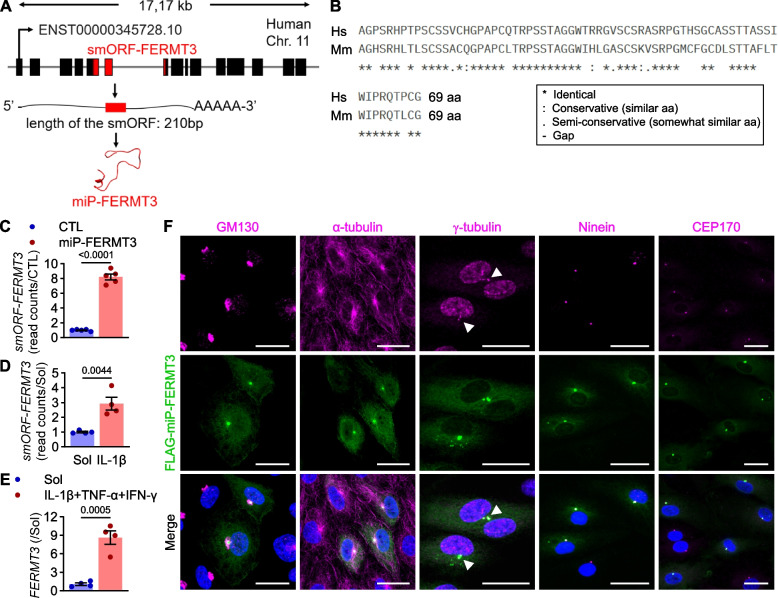
Location of smORF-FERMT3 and subcellular localization of miP-FERMT3. **A** Location of smORF-FERMT3 (red) in the *FERMT3* transcript and secondary structure prediction of miP-FERMT3 generated with AlphaFold3. **B** Sequence alignment of human and murine miP-FERMT3, generated with Clustal Omega. **C** *smORF-FERMT3* RNA expression (read counts) in endothelial cells expressing FLAG-miP-FERMT3 or miRFP647nano (CTL); *n* = 5 independent cell batches (unpaired, two-tailed Student’s t-test). **D** *smORF-FERMT3* RNA expression (RiboTag read counts) in endothelial cells following treatment with solvent (Sol) or interleukin-1β (IL-1β); *n* = 4 independent cell batches (unpaired, two-tailed Student’s t-test). **E** *FERMT3* RNA expression in endothelial cells treated with solvent (Sol) or IL-1β (20 ng/ml), TNF-α (10 ng/ml) and IFN-γ (10 ng/ml) for four days; *n* = 4 independent cell batches (unpaired, two-tailed Student’s t-test). **F** Confocal images showing FLAG-miP-FERMT3 together with GM130 (Golgi apparatus), α-tubulin (microtubules), γ-tubulin (centrosome, arrowheads), ninein or CEP170 (centriole subdistal appendages) in endothelial cells. Nuclei were stained with DAPI (blue). Similar results were obtained in 3–5 independent cell batches. Scale bar = 25 µm

The smORF encoding miP-FERMT3 spans exons 5–7 of the human *FERMT3* gene (+ chr 11:64,211,356–64,219,269) and comprises 210 bp. Computational structure prediction (AlphaFold3) indicated that miP-FERMT3 adopts a predominantly linear, intrinsically disordered conformation (Fig. 1A), and there is 69.6% homology between the human and the mouse miP-FERMT3 sequences (Fig. 1B). To investigate the subcellular localization of miP-FERMT3, adenoviruses were generated to express a FLAG-tagged miP-FERMT3 fusion protein in endothelial cells. Adenoviral transduction induced an approximately eightfold increase in *smORF-FERMT3* expression relative to control, which was comparable to the 3 to tenfold increase in *smORF-FERMT3/FERMT3* observed in endothelial cells treated with IL-1β or the combination of IL-1β, tumor necrosis factor-α (TNF-α) and interferon-γ (IFN-γ) (Fig. 1C-E). To identify the subcellular localization of the miP, FLAG immunofluorescence was combined with markers of the endoplasmic reticulum, Golgi apparatus, mitochondria, endosomes, lysosomes and centrosomes. This revealed that FLAG-miP-FERMT3 localized to the cytoplasm and accumulated near the Golgi apparatus where it colocalized with the centrosomal markers α-tubulin and γ-tubulin, as well as the centriole subdistal appendage markers ninein and CEP170 (Fig. 1F and Figure S1).

### miP-FERMT3 induces centrosome amplification and cell cycle arrest

**Fig. 2:**
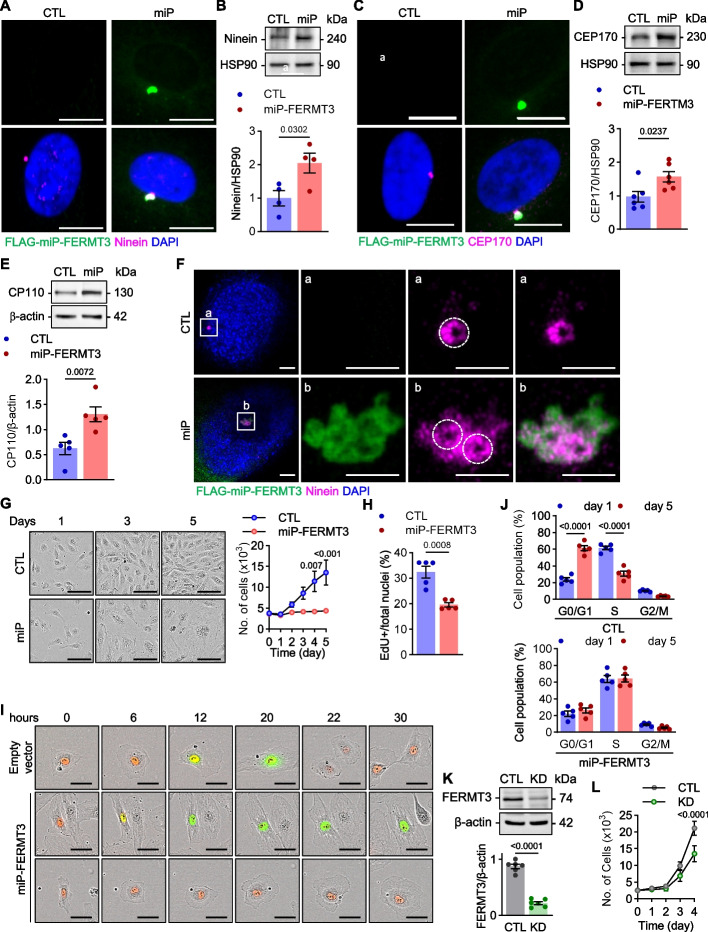
Impact of miP-FERMT3 expression on centrosomal proteins and cell-cycle progression. **A**-**B** Levels of ninein in endothelial cells expressing FLAG-miP-FERMT3 or EGFP (CTL) as determined by confocal microscopy (**A**) and SDS-PAGE (**B**). Similar results were obtained in 4–5 independent cell batches (unpaired, two-tailed Student’s t-test). Scale bar: 10 μm. **C-D** Levels of CEP170 in endothelial cells expressing FLAG-miP-FERMT3 or EGFP (CTL) as determined by confocal microscopy (**C**) and SDS-PAGE (**D**). Similar results were obtained in 4–6 independent cell batches (unpaired, two-tailed Student’s t-test). Scale bar: 10 μm. **E** Levels of CP110 in endothelial cells expressing FLAG-miP-FERMT3 or EGFP (CTL); *n* = 5 independent cell batches (unpaired, two-tailed Student’s t-test). **F** Representative expansion microscopy images showing coiled-coil images of ninein in endothelial cells expressing FLAG-miP-FERMT3 or EGFP (CTL). The regions highlighted as a and b are shown at higher magnification next to the first image. Similar results were obtained in 5 independent cell batches. Scale bars: 10 µm and 5 µm (a and b). **G** Representative images of growth factor-treated endothelial cells expressing FLAG-miP-FERMT3 or EGFP (CTL). The quantification reflects data from *n* = 5 independent cell batches (two-way ANOVA and Šidák’s multiple comparison). Scale bar: 100 µm. **H** EdU incorporation in cells expressing FLAG-miP-FERMT3 or EGFP (CTL); *n* = 5 independent cell batches (unpaired, two-tailed Student’s t-test). **I** Time course (up to 30 h) of changes in FUCCI fluorescence in endothelial cells expressing FLAG-miP-FERMT3 or empty vector. Red: G1 phase, yellow: G1/S, green: S/G2/M. Similar results were obtained in 4 additional independent cell batches. Scale bar: 50 µm. **J** Cell cycle progression (flow cytometry) of cells expressing FLAG-miP-FERMT3 or EGFP (CTL) showing DNA content distribution at day 1 and day 5 post-transduction; *n* = 5 independent cell batches (two-way ANOVA and Šídák's multiple comparisons test). **K** Expression of FERMT3 in endothelial cells transfected with a control small interfering RNA (CTL) or a small interfering RNA targeting human *FERMT3* (KD); *n* = 6 independent cell batches (unpaired, two-tailed Student’s t-test). **L** Growth factor-induced proliferation in endothelial cells transfected with a control small interfering RNA (CTL) or a small interfering RNA targeting human *FERMT3* (KD); *n* = 6 independent cell batches (two-way ANOVA and Šidák’s multiple comparison)

As cells progress through the cell cycle, centrosomes are duplicated and mature structurally to ensure proper mitosis. This process involves the expansion of the pericentriolar matrix and the sequential assembly of distal and subdistal appendage proteins at centrioles [14]. The centriole subdistal appendage protein ninein initially associates with the mother centriole before being distributed to the daughter centriole as centrosome duplication advances [15]. Another component of the centrosome, i.e. CEP170, is essential for the recruitment and stabilization of microtubules at the subdistal appendages [16]. Importantly, miP-FERMT3-expressing endothelial cells accumulated both ninein and CEP170 (Fig. 2A-D). Similarly, levels of the cyclin-dependent kinase (CDK) substrate CP110, a key regulator of centrosome duplication, were also elevated in miP-FERMT3-expressing cells (Fig. 2E). Expansion microscopy was used to better visualize structural changes in the subdistal appendages of centrioles. In naïve endothelial cells, ninein clustered into a ring, which is known to surround the centriole [17]. In miP-FERMT3-expressing cells ninein clusters formed multiple interconnected ring-like structures, indicative of centrosome duplication/amplification (Fig. 2F). These observations suggested that miP-FERMT3-expressing cells accumulated duplicated centrosomes, which is in turn indicative of incomplete cell cycle progression. Indeed, cell proliferation (Fig. 2G) and EdU incorporation (Fig. 2H), were markedly impaired in miP-FERMT3-expressing endothelial cells. These findings indicated that fewer cells progressed through the S/G2/M phases, an observation that was confirmed using a fluorescent ubiquitination-based cell cycle indicator (FUCCI) to visualise cycling cells. Indeed, we observed that miP-FERMT3 expression induced early cell cycle defects and cell cycle arrest during both the G1 (red) and S/G2/M (green) phases (Fig. 2I). Flow cytometry analyses of DNA content using Hoechst staining also revealed pronounced cell cycle arrest in miP-FERMT3-expressing cells (Fig. 2J). Even after five days of growth factor stimulation, miP-FERMT3-expressing cells remained in growth arrest with the majority (~ 60%) of cells in S phase, followed by G1 (~ 20%) and G2/M (~ 10%). As the smORF encoding miP-FERMT3 overlaps with the canonical FERMT3 ORF, small interfering RNA-mediated knockdown of *FERMT3* was performed to assess the consequence of depleting both the canonical protein and the miP. However, in line with the known role of FERMT3 in promoting endothelial cell proliferation and angiogenesis [18, 19], FERMT3 knockdown also inhibited cell proliferation (Fig. 2K-L).

### miP-FERMT3 expression elicits DNA damage and altered expression of cell-cycle related genes

**Fig. 3:**
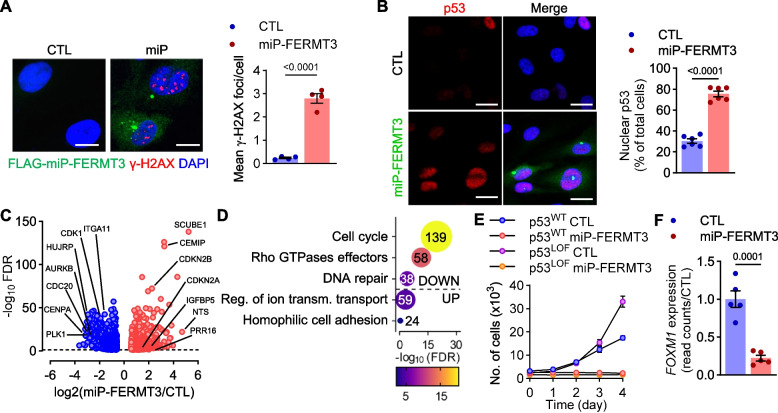
Impact of miP-FERMT3 expression on DNA damage, p53 nuclear accumulation and gene expression. **A** γ-H2AX foci in endothelial cells expressing FLAG-miP-FERMT3 or EGFP (CTL). *n* = 4 independent cell batches (unpaired, two-tailed Student’s t-test). Scale bar: 10 µm. **B** Confocal images showing p53 in endothelial cells expressing FLAG-miP-FERMT3 or EGFP (CTL); *n* = 6 independent cell batches (unpaired, two-tailed Student’s t-test). Scale bar = 25 µm. **C-D** Volcano plot (C) and gene set enrichment analysis (STRING with ranking – Reactome) (D) of upregulated (red) and downregulated (blue) genes in endothelial cells expressing FLAG-miP-FERMT3 or FLAG-miRFP670nano (CTL). Numbers inside bubble plot indicate # of genes/GO term; *n* = 5 independent cell batches. The dash line in C marks the significance threshold (FDR = 0.05). **E** Growth factor-induced proliferation of wild type RPE-1 cells expressing FLAG-miP-FERMT3 (p53^WT^ miP-FERMT3) or EGFP (p53^WT^ CTL) and p53 knockout RPE-1 cells expressing FLAG-miP-FERMT3 (p53^LOF^ miP-FERMT3) or EGFP (p53^LOF^ CTL); *n* = 5 independent cell batches (two-way ANOVA and Šidák’s multiple comparison). p53^WT^ CTL vs. p53^LOF^ CTL: day3 *p* = 0.0403, day4 *p* < 0.0001; p53^WT^ CTL vs. p53^WT^ miP-FERMT3: day2 *p* = 0.003, day3 and 4 *p* < 0.0001; p53^LOF^ CTL vs. p53.^LOF^ miP-FERMT3: day 2, 3 and 4 *p* < 0.0001. **F** RNA expression (read counts) of FOXM1 in endothelial cells expressing FLAG-miP-FERMT3 or miRFP647nano (CTL); *n* = 5 independent cell batches (unpaired, two-tailed Student’s t-test)

The dysregulation of centrosomal proteins and the resulting replicative stress contribute to genomic instability [20]. Activation of the DNA damage response promotes cell cycle arrest and senescence by stabilizing p53 and enhancing its nuclear accumulation through ataxia-telangiectasia mutated (ATM)/ataxia-telangiectasia and Rad3-related (ATR)-dependent signalling pathways [21, 22]. Therefore, we determined whether DNA damage occurred in miP-FERMT3-expressing cells. The presence of miP-FERMT3 did induce DNA damage as indicated by prominent γ-H2AX foci (Fig. 3A), a phenomenon that was concomitant with marked nuclear accumulation of p53. Indeed, approximately 75% of miP-FERMT3-expressing endothelial cells contained p53 in their nuclei (Fig. 3B). To determine the impact of miP-FERMT3 on transcription and identify affected pathways, we performed whole-transcriptome RNA sequencing of endothelial cells expressing the miP. This analysis identified 1410 miP-FERMT3-regulated genes (639 upregulated; 771 downregulated). Consistent with our functional data, many of the genes downregulated in miP-FERMT3-expressing cells were related to cell cycle progression. These included cyclin-dependent kinase 1 (*CDK1*), polo-like-kinase 1 (*PLK1*), aurora kinase B (*AURKB*), and DNA repair genes such as radiation sensitive protein 51 (*RAD51*), fanconi anemia group D2 (*FANCD2*), and exonuclease 1 (*EXO1*). In contrast, genes that were upregulated were implicated in cell cycle inhibition and senescence, including the cyclin-dependent kinase inhibitors *CDKN2A* and *CDKN2B* (Fig. 3C-D, Dataset 1). Even though miP-FERMT3 increased nuclear p53 levels, it did not induce the expression of classical p53-regulated genes such as *CDKN1A* (p21), *GADD45A* and *CCNG1*, indicating that p53 is unlikely to be the main effector target of the miP. To determine whether p53 was required for the miP-FERMT3-induced cell cycle arrest, we generated functional p53 knockouts (p53 loss-of-function, p53^LOF^) using CRISPR in RPE-1 cells. Wild-type and p53^LOF^ cells were adenovirally transduced to express miP-FERMT3 or EGFP as a control, and growth factor-induced proliferation was assessed. As expected, cell proliferation was significantly increased in p53^LOF^ cells compared to wild-type controls. Notably, however, expression of miP-FERMT3 induced cell cycle arrest irrespective of functional p53 signalling, indicating that the miP-FERMT3-driven arrest phenotype is largely p53-independent (Fig. 3E). Next, a transcription factor enrichment analysis (ChEA3 platform [23]) of all the differentially expressed genes predicted that forkhead box M1 (FOXM1) was the transcription factor most likely to be responsible for the regulation of a large proportion of the genes suppressed by miP-FERMT3 in endothelial cells (Dataset 2). Consistent with this prediction, FOXM1 was markedly downregulated in miP-FERMT3-expressing endothelial cells (Fig. 3F).

### miP-FERMT3 interacts with proteins involved in protein catabolism and enhances ubiquitination and proteasomal activity

**Fig. 4:**
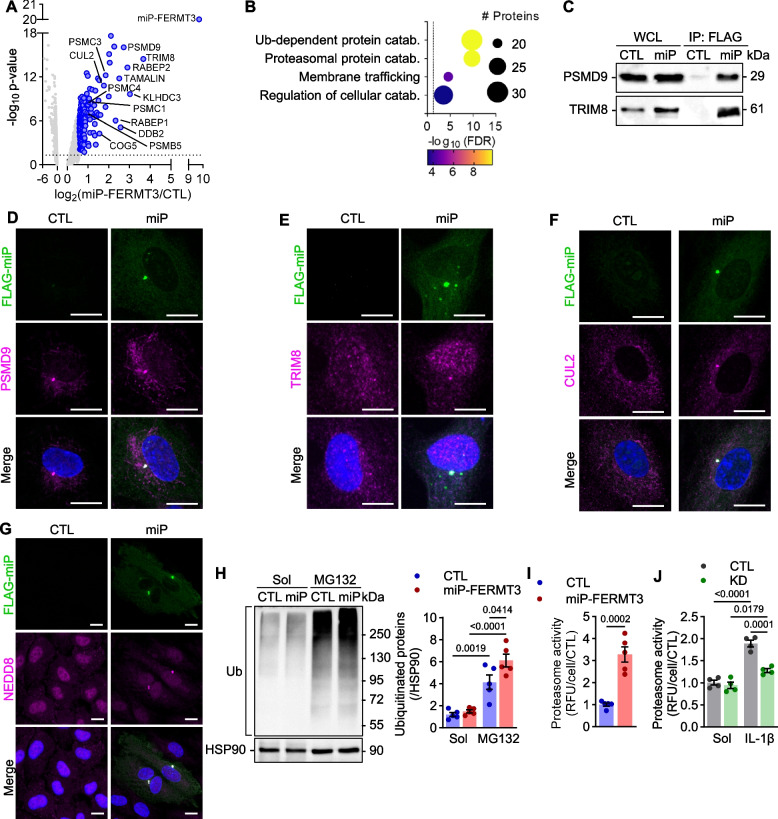
Interactome of miP-FERMT3 and effects on proteasomal activity. **A** Volcano plot showing proteins associated with FLAG-miP-FERMT3; *n* = 5 independent cell batches. The dashed line marks the significance threshold (*p* = 0.05). **B** GO term enrichment analysis (STRING) of FLAG-miP-FERMT3 interacting proteins. The dashed line marks the significance threshold (FDR = 0.05). **C** Co-immunoprecipitation (IP) of TRIM8 and PSMD9 with FLAG-miP-FERMT3 from endothelial cell lysates (input) expressing FLAG-miP-FERMT3 or EGFP (CTL). Similar results were observed in 4 independent cell batches. **D-G** Confocal images showing the colocalization of FLAG-miP-FERMT3 (FLAG-miP) with PSMD9 (**D**), TRIM8 (**E**), CUL2 (**F**) and NEDD8 (**G**). Nuclei were stained with DAPI (blue). Similar results were obtained in 3 additional independent cell batches. Scale bars = 10 µm. **H** Protein ubiquitination (Ub) in endothelial cells expressing FLAG-miP-FERMT3 or EGFP (CTL) and treated with solvent (Sol) or MG132 (10 μmol/L,16 h); *n* = 5 independent cell batches (two-way ANOVA and Šídák's multiple comparison). **I** Proteasome activity in endothelial cells expressing FLAG-miP-FERMT3 or EGFP (CTL); *n* = 5 independent cell batches (unpaired, two-tailed Student’s t-test). **J** Proteasomal activity in endothelial cells transfected with a control small interfering RNA (CTL) or a small interfering RNA targeting human *FERMT3* (KD) and treated with solvent (Sol) or interleukin-1β (IL-1β, 20 ng/ml for 16 h); *n* = 4 independent cell batches (two-way ANOVA and Šídák's multiple comparison)

Microproteins often exert their biological function as components of macromolecular complexes [24–26]. To identify proteins that interacted with miP-FERMT3, the FLAG-tagged fusion protein was expressed in endothelial cells, immunoprecipitated and the recovered complexes were subjected to mass spectrometry. This approach identified 180 proteins enriched by at least 1.5-fold in miP-FERMT3 immunoprecipitates, most of which were related to ubiquitin and proteasome-dependent protein catabolic processes (Fig. 4A-B, Dataset 3). Among the latter were 23 regulatory and catalytic subunits of the 26S proteasome and 6 E3 ubiquitin ligases, including 26S proteasome non-ATPase regulatory subunit 9 (PSMD9) and tripartite motif containing 8 (TRIM8). PSMD9 is a ubiquitously expressed proteasomal chaperone that facilitates the assembly of the 26S proteasome and contributes to proteostasis in mammalian cells [27], and TRIM8 is a member of the RING-type E3 ubiquitin ligase family that regulates a broad range of cellular processes [28]. The interaction between miP-FERMT3, PSMD9 and TRIM8 was validated by co-immunoprecipitation and immunoblotting (Fig. 4C) and occurred predominantly at the centrosome (Fig. 4D-E). Another miP-FERMT3-interacting protein involved in protein catabolism was cullin-2 (CUL2). This protein serves as a scaffold of Cullin–RING ligase complexes, which comprise a cullin scaffold, a RING-finger protein, adaptor proteins and a substrate-recognition module [29]. This macromolecular complex mediates the ubiquitination of numerous target proteins and confocal microscopy confirmed the colocalization of miP-FERMT3 and CUL2 (Fig. 4F). The activity of cullin is tightly regulated by its post-translational modification with the ubiquitin-like molecule NEDD8, in a process referred to as neddylation [30]. In control endothelial cells, NEDD8 appeared to be distributed throughout the nucleus, but in cells expressing miP-FERMT3 it was highly concentrated in distinct spots that co-localized with the FLAG-tagged miP (Fig. 4G). As our observations suggested that the miP may modulate ubiquitin-dependent processes, we next determined whether protein ubiquitination was altered in miP-FERMT3-expressing cells. Global cellular ubiquitination was consistently increased in miP-FERMT3-expressing cells treated with MG132 to inhibit proteasomal degradation (Fig. 4H). Notably, proteasomal activity, assessed by determining the degradation of a specific fluorogenic substrate, was also significantly enhanced in miP-FERMT3-expressing cells (Fig. 4I). As miP-FERMT3 was identified in cells treated with IL-1β, we determined the impact of the cytokine on proteasomal activity. This revealed a twofold increase in proteasomal activity in IL-1β-treated cells that was significantly reduced after FERMT3/miP-FERMT3 knockdown (Fig. 4J).

### Impact of miP-FERMT3 on p21 turnover

**Fig. 5:**
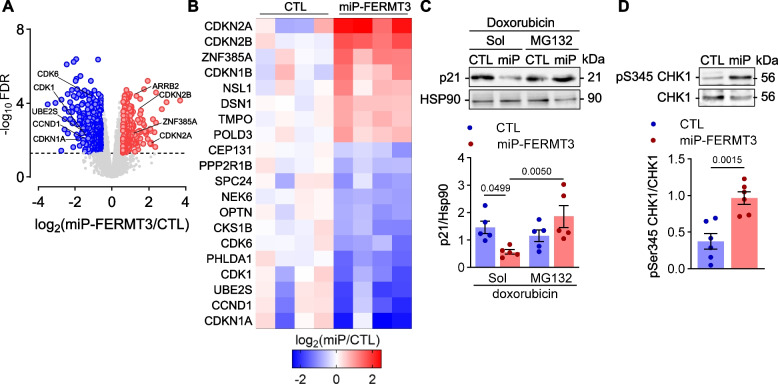
Effect of miP-FERMT3 on p21 turnover. **A** Volcano plot of upregulated (red) and downregulated (blue) proteins in endothelial cells expressing FLAG-miP-FERMT3 or FLAG-miRFP670nano (CTL). The dash line marks the significance threshold (FDR = 0.05). **B** Significantly (FDR ≤ 0.05) upregulated (red) and downregulated (blue) cell cycle-related proteins in endothelial cells expressing FLAG-miP-FERMT3 or FLAG-miRFP670nano (CTL); n = 4 independent cell batches. **C** Levels of p21 in endothelial cells expressing FLAG-miP-FERMT3 or EGFP (CTL) treated with doxorubicin (250 nmol/L) and either MG132 (10 μmol/L) or solvent (Sol) for 16 h; *n* = 5 independent cell batches (two-way ANOVA and Šídák's multiple comparison). **D** Phosphorylation of CHK1 on Ser345 in endothelial cells expressing FLAG-miP-FERMT3 or EGFP (CTL); *n* = 6 independent cell batches (unpaired, two-tailed Student’s t-test)

Next, we sought to identify proteins whose expression was altered in the presence of miP-FERMT3 and that could account for the observed DNA damage and cell cycle arrest. Multiplexed whole-cell quantitative proteomics analysis revealed that 198 proteins were upregulated and 354 proteins were downregulated (FDR ≤ 0.05, fold change ≥ ± 1.5) in miP-FERMT3-expressing endothelial cells (Fig. 5A, Dataset 4). Intersection of the differentially regulated proteins with a reference list of proteins involved in cell cycle (Reactome stable identifier: R-HSA-1640170) corroborated the upregulation of cell cycle inhibitors such as CDKN2A, CDKN2B and CDKN1B and the downregulation of key proteins involved in cell cycle progression, such as CDK1, CDK6 and CCND1 (Fig. 5B). CDKN1A (p21) was the most strongly downregulated protein in miP-FERMT3-expressing endothelial cells, a finding that was initially unexpected as high p21 levels are typically associated with cell cycle arrest [31]. To further investigate the link between miP-FERMT3 and p21 dynamics, DNA damage was induced using doxorubicin and p21 protein turnover was assessed. Treatment with doxorubicin elicited the anticipated robust accumulation of p21, a response that was significantly attenuated in miP-FERMT3-expressing endothelial cells (Fig. 5C). Inhibition of proteasome-mediated protein degradation restored p21 levels in miP-FERMT3-expressing endothelial cells, indicating a link between the miP and increased p21 turnover. Given that p21 is required for proper exit from S phase [32, 33], a decrease in its abundance in miP-FERMT3-expressing endothelial cells could promote replicative stress and subsequent DNA damage. In response to impaired DNA replication, cell cycle arrest is mediated by activation of the ATR- checkpoint kinase 1 (CHK1) pathway, specifically through the ATR-dependent phosphorylation of CHK1 on Ser345 [34]. Consistent with the presence of replicative stress and S phase arrest, miP-FERMT3-expressing endothelial cells exhibited a pronounced increase in CHK1 phosphorylation on Ser345 (Fig. 5D).

### Expression of miP-FERMT3 induces cellular senescence

**Fig. 6:**
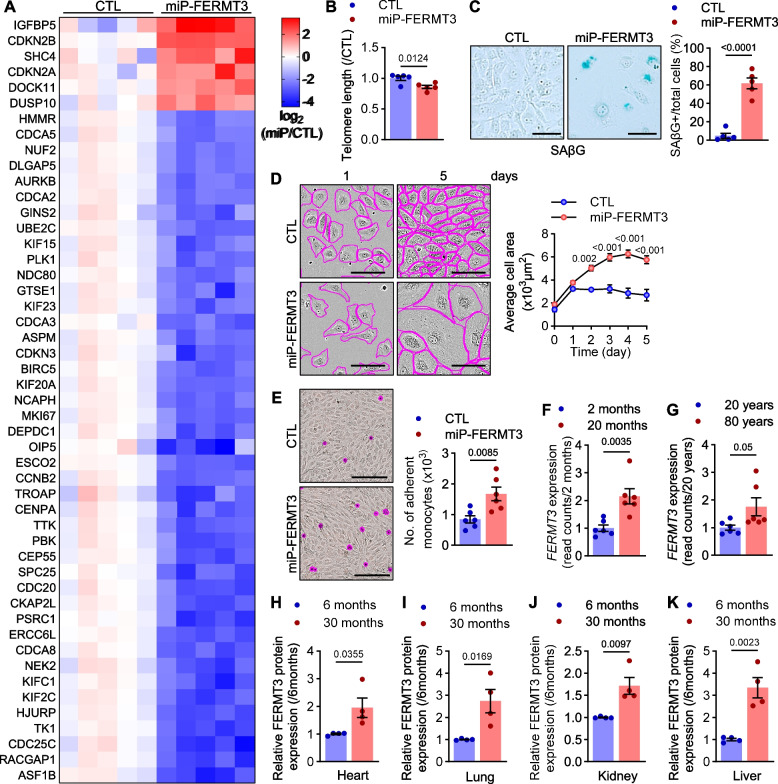
Impact of miP-FERMT3 on cellular senescence. **A** Senescence-associated genes significantly (FDR ≤ 0.05) altered by FLAG-miP-FERMT3 versus FLAG-miRFP670nano (CTL) expression; *n* = 5 independent cell batches. **B** Telomere length in cells expressing FLAG-miP-FERMT3 or EGFP (CTL); *n* = 5 independent cell batches (unpaired, two-tailed Student’s t-test). **C** SAβG in cells expressing FLAG-miP-FERMT3 or EGFP (CTL); *n* = 6 independent cell batches (unpaired, two-tailed Student’s t-test). Scale bar: 100 µm. **D** Average cell area in cells expressing FLAG-miP-FERMT3 or EGFP (CTL); *n* = 6 independent cell batches (two-way ANOVA and Šidák’s multiple comparison). Scale bar: 100 µm. **E** Adhesion of monocytes (magenta) to endothelial cell monolayers following incubation with conditioned medium from cells expressing FLAG-miP-FERMT3 or EGFP (CTL); *n* = 6 independent cell batches (unpaired, two-tailed Student’s t-test). Scale bar: 200 µm. **F** *FERMT3* transcript expression (read counts) in cardiac endothelial cells from 2- versus 20-month-old mice; n = 6 mice/group (unpaired, two-tailed Student’s t-test). **G** *FERMT3* transcript expression (read counts) in mesenteric artery endothelial cells from 20- versus 80-year-old subjects; *n* = 6 individuals/group (unpaired, two-tailed Student’s t-test). **H–K** FERMT3 protein levels in hearts (**H**), lungs (**I**), kidneys (**J**) and livers (**K**) from 6- versus 30-month-old mice; *n* = 4 mice/group (unpaired, two-tailed Student’s t-test)

Endothelial senescence is characterized by irreversible cell-cycle arrest and loss of proliferative potential [35]. To investigate whether miP-FERMT3 merely prevented proliferation or also induced senescence, we intersected the differentially regulated genes in miP-FERMT3-expressing endothelial cells with a curated dataset of senescence-associated genes using Senescent Cell Identification (SenCID) [36]. This analysis revealed a robust senescence-associated gene expression signature in miP-FERMT3-expressing cells (Fig. 6A). Further validation of the induction of senescence was provided by estimating telomere length and senescence-associated β-galactosidase (SAβG) activity. Indeed, miP-FERMT3 expression resulted in a significant shortening of telomeres, as quantified by monochrome multiplex qPCR (Fig. 6B). Moreover, approximately 60% of the miP-FERMT3-expressing cells expressed SAβG within 7 days of transduction (Fig. 6C). This is a significant finding as repetitive passaging of cells seeded at low density for up to 10 times is generally required for human endothelial cells to express SAβG [37] and the cells studied were passaged maximally six times. Indeed, SAβG was largely undetectable in the cells transduced with the control virus. Live cell imaging revealed that already two days after transduction, miP-FERMT3 expression had induced a clear change in endothelial cell morphology. The cells expressing the miP lost their typical cobblestone morphology and increased in size, a phenomenon that became more pronounced with time (Fig. 6D). Cellular senescence has been associated with a change in the secretome that includes the release of pro-inflammatory mediators [38, 39]. In line with this, the application of culture medium from miP-FERMT3-expressing cells to untreated endothelial cells induced inflammatory cell activation as evidenced by increased monocyte adhesion compared with cells treated with culture medium from EGFP-expressing cells (Fig. 6E).

The vasculature is among the first systems to “age” and has even been proposed to orchestrate systemic aging [10, 40]. To determine whether the expression of the *FERMT3* transcript, which hosts the smORF encoding miP-FERMT3, is affected by aging, we interrogated publicly available datasets that compared endothelial cell transcriptomes from young and aged mice and humans [37, 41]. This revealed higher *FERMT3* expression in endothelial cells from 20-month-old versus 2-month-old mice (Fig. 6F). Similarly, endothelial cells from 80-year-old humans expressed higher *FERMT3* expression than those from 20-year-old subjects (Fig. 6G). Data from a recent aging mouse proteome atlas [42], confirmed these findings as FERMT3 protein was significantly more abundant in the heart, lungs, liver and kidneys of 30 month versus 6 month old mice (Fig. 6H-K).

## Discussion

In this study, we characterized miP-FERMT3, a previously unannotated miP translated from the *FERMT3* transcript, but in a different frame, in inflammation-activated human endothelial cells. Functionally, miP-FERMT3 expression rapidly induced cell cycle arrest, centrosome amplification and DNA damage, leading to endothelial senescence and a paracrine pro-inflammatory phenotype. At the molecular level, miP-FERMT3 associated with centriole subdistal appendages, where it interacted with components of the protein catabolic machinery, enhancing neddylation, ubiquitination and proteasomal activity. As a direct consequence, miP-FERMT3-expressing cells exhibited a higher turnover of p21, contributing to genomic instability, arrest is S phase and induction of senescence-associated transcriptional programs. Notably, *FERMT3* expression was elevated in aged mouse and human endothelial cells, which hints at a potential role of miP-FERMT3 in vascular senescence and aging.

The location of a miP in a cell frequently gives hints about its likely cellular function. Indeed, miPs that associate with membrane receptors modulate downstream signal transduction pathways, while miPs that regulate cell metabolism have been localized to mitochondria [43–45]. In endothelial cells, FLAG-miP-FERMT3 was diffusely distributed through the cytosol as well as in distinct hotspots close to the nucleus. The latter were identified as centrosomes by virtue of colocalization with the centrosomal markers α- and γ-tubulin, as well as the centriole subdistal appendage protein markers ninein and CEP170. Centrosomes play an essential role in the control of cell cycle progression, cytoskeletal organization, the DNA damage response and proteostasis [11, 13, 43]. During S phase, the centrosome undergoes duplication accompanied by expansion of the pericentriolar matrix and the assembly of distal and subdistal appendage proteins, including ninein and CEP170 [14]. In addition, ninein functions as a component of the centrosome linker complex, which maintains centriole cohesion until its dissolution in late G2 [46, 47]. In miP-FERMT3-expressing cells, there was an accumulation of ninein aggregates as well as an increase in CEP170 and CP110 levels, indicating erroneous centrosome duplication that was consistent with a failure to progress beyond S phase. Defects in centrosome duplication have been shown to elicit a robust senescence response [48], in line with the rapid onset of senescence observed in miP-FERMT3-expressing endothelial cells.

The FLAG-miP-FERMT3 in centrosomes physically interacted with multiple proteins implicated in ubiquitin- and proteasome-dependent protein catabolism. Among the top miP-FERMT3-interacting proteins were PSMD9, a regulatory subunit of the 19S particle of the 26S proteasome complex [49], CUL2, a scaffold protein of cullin-RING E3 ubiquitin ligase complexes [50] and TRIM8, an E3 ubiquitin ligase [51]. RING-finger E3 ligases, including the cullin-RING family [52], represent the largest and most diverse class of E3 enzymes and are regulated by post-translation modification with NEDD8 [30]. Importantly, NEDD8 accumulated at centrosomes in miP-FERMT3-expressing cells, implying activation of cullin-RING E3 ligases in this compartment. The association of miP-FERMT3 with PSMD9 and CUL2 pointed to a potential role of the miP in regulating E3 ligase activity and proteasome-dependent protein turnover. Although miP-FERMT3 expression had only a modest impact on global cellular protein ubiquitination, it was associated with a significant increase in proteasomal activity. Stimulation with IL-1β, which upregulates *FERMT3* expression, elicited a comparable increase in proteasomal activity. This effect was largely attributable to either FERMT3, miP-FERMT3 or both, as it was strongly attenuated in FERMT3-deficient cells. However, given the lack of evidence linking FERMT3 to protein catabolism, it is plausible that the observed increase in proteasomal activity in IL-1β–treated endothelial cells is primarily mediated by miP-FERMT3.

Consistent with enhanced protein degradation, about twice as many proteins were down- versus up-regulated in miP-FERMT3-expressing endothelial cells. Of the altered proteins involved in cell cycle regulation, p21 was most affected by miP-FERMT3 expression. p21 levels are tightly regulated in a cell cycle-dependent manner [53, 54], and its levels are actively supressed by cullin-RING E3 ligase-mediated proteasomal degradation to enable DNA replication during S phase [32, 55]. Thereafter, the accumulation of p21 is required to restrain CDK activity and allow S phase exit. On the basis of our findings, it is tempting to suggest that elevated proteasomal activity in miP-FERMT3-expressing cells prevents p21 accumulation, thereby impairing orderly S phase completion and cell cycle progression. Consistent with such an effect is that while the degradation of p21 during S phase is required to avoid replication defects [56], its sustained loss has been shown to promote deregulated replication licensing and genomic instability [32, 33]. Failure to restore p21 levels at the end of S phase is therefore likely to lead to persistent CDK activity, replication stress and DNA damage, as was the case in miP-FERMT3 expressing endothelial cells as evidenced by increased γ-H2AX foci. Under such conditions, cell cycle arrest can be enforced independently of p53 through activation of checkpoint pathways, e.g., the ATR-CHK1 axis [34, 57]. Consistent with the activation of a DNA damage and replication stress response during S phase, miP-FERMT3-expressing cells exhibited increased ATR-dependent CHK1 phosphorylation. As this checkpoint response operates largely at the post-translational level, it can sustain cell cycle arrest in the absence of a classical transcriptional p21 response, resulting in a stress-induced, rather than a canonical p53-p21-driven arrest state [21, 58, 59]. In line with such a chain of events, miP-FERMT3 expression induced cell cycle arrest also in cells with impaired p53 signalling, providing a potential explanation as to why increased nuclear p53 accumulation in miP-FERMT3-expressing endothelial cells did not translate into induction of classical p53 target genes, including p21. Our findings fit well with previous reports indicating that replication stress can stabilize nuclear p53 without fully activating its transcriptional program, thereby functionally uncoupling p53 accumulation from p21 induction [59, 60]. Transcription factor profiling identified FOXM1 as a key potential regulator underlying the decreased expression of cell cycle-associated genes and upregulation of cell cycle inhibitors in endothelial cells expressing miP-FERMT3. FOXM1 promotes cell cycle progression by driving the expression of genes involved in DNA replication, mitosis and genomic stability, and is negatively regulated by p53 [61, 62]. Reduced FOXM1 is also a hallmark of cellular senescence, leading to irreversible cell cycle arrest and centrosome amplification through defective G1/S and G2/M transitions [63–66], consistent with the cellular features observed in miP-FERMT3-expressing endothelial cells.

Manipulation of the internal ORF encoding miP-FERMT3 inevitably also affects the host transcript, making it difficult to define the specific contribution of endogenous miP-FERMT3 to cell cycle progression. FERMT3 downregulation also impaired cell proliferation, consistent with previous reports that FERMT3 promotes endothelial cell proliferation and angiogenesis [18, 19]. These findings suggest that miP-FERMT3 and FERMT3 exert opposite effects on endothelial cell proliferation, with miP-FERMT3 eliciting a markedly stronger inhibitory effect. Thus, FERMT3/miP-FERMT3 expression must be tightly regulated to maintain proper control of cell cycle progression.

The consequence of miP-FERMT3-induced replicative stress was a rapid phenotypic change consistent with cellular senescence, including increased cell size, the expression of SAβG, telomere attrition and the secretion of factors that promoted endothelial activation and increased monocyte adhesion to the cell surface. Given the strong link between vascular senescence and aging, we sought to determine whether expression of miP-FERMT3 was altered in aging in vivo. Direct assessment of endogenous miP-FERMT3 was not feasible because no tools are available for its specific detection. Moreover, interrogation of publicly available human and murine ribosome profiling datasets was not informative, as the low sequencing depth and limited coverage of the *FERMT3* transcript precluded a reliable detection of smORF-FERMT3 translation. Although FERMT3 transcript and protein abundance increased with age in human and murine endothelial datasets, these measures cannot be used to infer endogenous miP-FERMT3 levels in the absence of evidence for coordinated regulation of translation from the internal ORF. Thus, whether miP-FERMT3 is similarly upregulated during vascular aging remains an open question.

Prior proteogenomic evidence supports translation of the smORF from the FERMT3 locus and miP-FERMT3 was detected by mass spectrometry-based proteomics [7]. However, an important limitation of the present study is the lack of orthogonal validation of the expression, regulation and cellular distribution of the endogenous miP-FERMT3. Accordingly, the mechanistic conclusions presented here are based predominantly on a gain-of-function approach using FLAG-tagged miP-FERMT3. While these experiments provide insight into the potential cellular consequences of miP-FERMT3 expression, they do not fully establish its pathophysiological relevance.

Taken together, this study demonstrates that miP-FERMT3 modulates the ubiquitin–proteasome system and elicits p53-independent cell cycle arrest and cellular senescence. The upregulation of the miP in the context of inflammation may contribute to the onset of vascular senescence that is a hallmark of aging.

## Methods

### Cell culture

Human umbilical vein endothelial cells were isolated and cultured as described previously [67, 68] and used between passage 4–6. The use of human material in this study complies with the principles outlined in the Declaration of Helsinki (World Medical Association, 2013), and the isolation of endothelial cells was approved in written form by the ethics committee of the Goethe-University. THP-1 monocytic cells were obtained from the American Type Culture Collection (LGC Standards; Hamburg, Germany) and cultured in RPMI-1640 containing 2 mmol/L glutamine, 10 mmol/L HEPES, 1 mmol/L sodium pyruvate, 4.5 g/L glucose, 1.5 g/L sodium bicarbonate and 10% foetal calf serum. AD-293 cells were cultured as described previously [69]. RPE-1 cells were originally obtained from ATCC (CRL-4000), passaged in DMEM/F12 media containing 10% serum, 1% pen/strep, and 0.01 mg/ml hygromycin B (Capricorn Scientific, HYG-H). RPE-1 p53^LOF^ cells were generated by cloning the TP53-targeting gRNA (5’-CCAGTTGCAAACCAGACCTC-3’) into pLenti-Guide and transducing wild-type RPE-1 cells with the corresponding infectious lentiviral particles. Selection of p53^LOF^ cells was performed 96 h post-transduction by culture in the presence of 1 µmol/L Nutlin-3a (Selleck, S8059). lentiGuide-Puro was a gift from Feng Zhang (Addgene plasmid # 52,963; http://n2t.net/addgene:52963; RRID: Addgene_52963). All cell cultures tested negative for mycoplasma contamination. Cells were maintained in a humidified incubator at 37 °C with 5% CO₂.

Cells were treated with 10 μmol/L MG132 (Cat. # M7449; Merck, Darmstadt, Germany) with or without 250 nmol/L doxorubicin (Cat. # D5220; Merck, Darmstadt, Germany) or 0.1% dimethyl sulfoxide (DMSO) as solvent control in endothelial cell growth medium 2 (ECGM2; PromoCell, Heidelberg, Germany) for 16 or 24 h at 37 °C.

Cells were treated with solvent control or 20 ng/ml IL-1β (Cat. # 200-01B; Peprotech, New Jersey, USA) alone or in combination with 10 ng/ml TNF-α (Cat. # 300-01A; Peprotech, New Jersey, USA), and 10 ng/ml IFN-γ (Cat. # 300–02; Peprotech, New Jersey, USA) for 16 h or four days.

### Generation of adenoviruses, lentiviruses and cell transduction

#### Adenovirus generation and transduction

The adenoviral vector used to overexpress FLAG-tagged miP-FERMT3 was constructed and packaged by VectorBuilder. Briefly, the sequence encoding the FLAG tag (GACTACAAAGACGATGACGACAAG) was inserted at the 5’ end of the human smORF-FERMT3 sequence (GCCGGCCCCAGCCGCCACCCGACCCCCTCCTGCTCCAGCGTCTGCCACGGCCCAGCTCCCTGTCAGACAAGACCCAGCTCCACAGCAGGTGGCTGGACTCGTCGCGGTGTCTCATGCAGCAGGGCATCAAGGCCGGGGACGCACTCTGGCTGCGCTTCAAGTACTACAGCTTCTTCGATTTGGATCCCAAGACAGACCCCGTGCGGCTGA), preceded by Kozak sequence (GCCACC) and ATG start codon. The restriction sites AbsI and SgrDI were placed on the 5’ and 3’ end on of the insert, respectively (VectorBuilder ID: VB230707-1073jqc). The insert was cloned into the mammalian gene expression adenoviral vector pAd5 under the cytomegalovirus (CMV) promoter. DNA sequencing confirmed correct direction of the insert and lack of mutations. The adenoviral vector and replication incompetent adenoviruses used to overexpress FLAG-tagged miRFP670nano or EGFP were also constructed and packaged by VectorBuilder as described above (VectorBuilder ID: VB230223-1677bmd, VB010000-9299hac). FLAG-miRFP670nano was used as control as it represents smallest near infra-red fluorescent protein. Alternatively, for experiments involving the Fluorescent Ubiquitination-based Cell Cycle Indicator, adenoviruses carrying an empty pAdShuttle-CMV vector were generated as described [70]. Generation and expansion of all replication incompetent adenoviruses was carried out by transfection of the packaging cell line AD-293. Human endothelial cells (passage 4, 90% confluency) were starved of serum in endothelial cell basal medium (EBM, Cat. # C-22211; PromoCell, Heidelberg, Germany) containing 0.1% bovine serum albumin (Cat. # A8412; Sigma-Aldrich; Darmstadt, Germany) and infected with adenoviruses (250 MOI) overnight. On the following day, the medium was replaced with ECGM2 supplemented with 10% foetal calf serum (Sigma-Aldrich; Darmstadt, Germany).

#### Lentivirus generation and transduction

Lentiviruses to express the Fluorescent Ubiquitination-based Cell Cycle Indicator were generated as described [71]. Human endothelial cells were transduced with two lentiviruses to express mCherry-hCdt1 and mAG-hGeminin for 24 h in the presence of 10 µg/mL polybrene (Cat. # sc-134220; Santa Cruz Biotechnology, Heidelberg, Germany). Following transduction, cells were cultured for an additional 24 h before further experiments.

### Immunofluorescence

Cells grown on 8 well chamber slides (Ibidi, Martinsried, Germany) were washed with phosphate buffered saline (PBS) containing 0.8 mmol/L CaCl_2_ and 1.4 mmol/L MgCl_2_. For mitochondrial staining, cells were incubated with 100 nmol/L MitoTracker® Red CMXRos (Cat. # M7512, ThermoFisher Scientific; Darmstadt, Germany) for 30 min in a humidified incubator at 37ºC. Then cells were fixed in ice cold methanol (Sigma-Aldrich; Darmstadt, Germany) or 4% paraformaldehyde (ThermoFisher Scientific; Darmstadt, Germany) and incubated in blocking/permeabilization buffer containing 5% horse serum and 0.1% Triton X-100 (Cat. 3051.2, Carl Roth, Karlsruhe, Germany) in PBS for 30 min at room temperature, followed by incubation with anti-calnexin (Cat. # C4731, Sigma-Aldrich; Darmstadt, Germany), anti-CEP170 (Cat. # 27,325–1-AP, Proteintech; Planegg-Martinsried, Germany), anti-CUL2 (Cat. # 67,175–1-IG, ThermoFisher Scientific, Darmstadt, Germany), anti-EEA1 (Cat. # 3288, Cell Signaling Technology; Leiden, Netherlands), anti-FLAG (Cat. # F3165 or # F7425, Sigma-Aldrich; Darmstadt, Germany), anti-GM130 (Cat. # ab52649, Abcam; Amsterdam, Netherlands), anti-LAMP1 (Cat. # 9091, Cell Signaling Technology; Leiden, Netherlands), anti-NEDD8 (Cat. # ab81264, Abcam; Amsterdam, Netherlands), anti-ninein (Cat. # ab4447, Abcam; Amsterdam, Netherlands), anti-NOGO-B (Cat. # ab47085, Abcam; Amsterdam, Netherlands), anti-p53 (Cat. # 2527S; Cell Signaling Technology; Leiden, Netherlands), anti-PSMD9 (Cat. # ab233154, Abcam; Amsterdam, Netherlands), anti-TRIM8 (Cat. # PA5-142,041, Invitrogen; Darmstadt, Germany), anti-α-tubulin (Cat. # 2125, Cell Signaling Technology; Leiden, Netherlands), anti-γ-H2AX (Cat. # ab81299, Abcam; Amsterdam, Netherlands), anti-γ-tubulin (Cat. # ab11317, Abcam; Amsterdam, Netherlands) in 0.5% horse serum and 0.01% Triton X-100 in PBS for 2 h at room temperature. Cells were then incubated with donkey anti-mouse Alexa Fluor 488 (Cat. # A-21202; Invitrogen, Darmstadt, Germany) and donkey anti-rabbit Alexa Fluor 555 (Cat. # A-32794; Invitrogen, Darmstadt, Germany) in PBS for 1 h at room temperature. Nuclei were stained using 4′,6-diamidino-2-phenylindole (DAPI) (Cat. # A1001, Applichem GmbH; Darmstadt, Germany). Finally, cells were overlaid with mounting medium containing 50% (v/v) glycerol (Cat. # 3783.1, Carl Roth, Karlsruhe, Germany) and dithiothreitol (DTT, 1 mol/L) (Cat. # A2948, Applichem GmbH; Darmstadt, Germany). Images were taken using an SP8 (Leica, Wetzlar, Germany) or LSM-780 confocal microscope (Zeiss, Jena, Germany) and LAS AF lite software (Leica) or ZEN software (Zeiss).

The mean fluorescence intensity (MFI) of p53 and mean γ-H2AX foci were quantified using ImageJ (version 1.54d) software. MFI values for p53 were normalized to the MFI of DAPI to account for nuclear area, and results were expressed as the percentage of total nuclear p53. The number of p53 positive cells that were FLAG-miP-positive or FLAG-miP-negative were quantified and expressed as percentage of p53 cells. For each experimental cell batch, 3–5 different fields were imaged and analysed for quantification. For quantification of γ-H2AX foci, at least 200 cells per batch were analysed, and the mean number of γ-H2AX foci per cell was calculated.

### Expansion microscopy

Preparation for expansion microscopy was done as described previously [72, 73]. Briefly, cultured cells for expansion microscopy were fixed on 13 mm coverslips ice cold methanol for 15 min. Cells were immunostained with anti-Ninein (Cat. # ab4447; Abcam; Amsterdam, Netherlands), and anti-FLAG (Cat. # F3165; Sigma-Aldrich; Darmstadt, Germany) antibodies for 3 days and then donkey anti-mouse Alexa Fluor 488 (Cat. # A-21202; Invitrogen, Darmstadt, Germany), donkey anti-rabbit Alexa Fluor 555 (Cat. # A-32794; Invitrogen, Darmstadt, Germany) and DAPI in PBS for 2 days. Stained cells on coverslips were transferred to 35 mm MatTek dishes (P35G-1.0–14-C, MatTek) and incubated with 10 mg/ml Acryloyl-X/DMSO in PBS (1:100) for 3 h at room temperature. Samples were washed twice with PBS for 15 min. Samples were subsequently incubated with a freshly prepared gelling solution composed of Stock X, tetramethylethylenediamine, ammonium persulfate, and distilled water in a 47:1:1:1 (v/v/v/v) ratio. Stock X contained 0.914 mol/L sodium acrylate, 0.352 mol/L acrylamide, 9.7 mmol/L N,N′-methylenebisacrylamide, 2 mol/L NaCl, and 1 × PBS in water. Then, samples were covered with a glass 15 mm coverslip and incubated at 37 °C for 2 h. After the gel polymerized, the coverslips were removed and the gel was incubated with an aqueous digestion buffer containing 0.5% Triton X-100, 1 mmol/L EDTA disodium pH 8.0, 50 mmol/L Tris–HCl pH 8.0, 800 mmol/L NaCl and 8 U/ml Proteinase K on a shaker overnight at room temperature. After removal of the digestion buffer, gel samples were incubated with distilled water 4 times for 20 min. Samples were embedded in 4% low-melting point agarose in water in a 35 mm dish containing a polymer coverslip (81,151, Ibidi). Images were taken using a confocal microscope (LSM-780; Zeiss, Jena, Germany) and ZEN software (Zeiss).

### FERMT3 knockdown

Human endothelial cells (passage 3) were seeded at a confluency of 70%. Twenty-four hours after seeding, transfection was performed for 5 h using 20 nmol/L siRNAs targeting the human *FERMT3* sequence 5’-CACTGACTTTGTGCAGGCCAA-3’ (SI05065473, sense: 5’-CUGACUUUGUGCAGGCCAATT-3’, antisense: 5’-UUGGCCUGCACAAAGUCAGTG-3’, Qiagen, Germany) or 20 nmol/L negative control siRNA (#1,022,076, Qiagen, Germany). The siRNA:Lipofectamine RNAiMAX transfection reagent (Cat. # 13,778,150, Invitrogen) ratio was 1:0.3 according to the manufacturer’s instructions. The medium was then replaced with fresh ECGM2 and transfected cells were used for assays up to 6 days after transfection.

### Cell lysis and immunoblotting

Samples were lysed in ice-cold radioimmunoprecipitation assay buffer (50 mmol/L Tris HCl pH 7.5, 150 mmol/L NaCl, 25 mmol/L NaF, 10 mmol/L Na_4_P_2_O_7_, 1% Triton X-100 and 0.5% sodium deoxycholate) supplemented with 0.1% sodium dodecyl sulfate (SDS) and protease and phosphatase inhibitors. Protein concentrations were determined using the Bradford assay, and detergent-soluble proteins were solubilized in sample buffer containing 2% SDS, 1% β-mercaptoethanol and 0.005% bromophenol, separated by SDS-PAGE and subjected to immunoblotting. Membranes were incubated with anti-ninein (AB4447, Abcam; Amsterdam, Netherlands), anti-CEP170 (Cat. # 27,325–1-AP, Proteintech; Planegg-Martinsried, Germany), anti-TRIM8 (Cat. # ab316149, Abcam; Amsterdam, Netherlands), anti-K48 polyubiquitin (Cat. # 8081, Cell Signaling Technology; Leiden, Netherlands), anti-CP110 (Cat. # 12,780–1-AP, ThermoFisher Scientific; Darmstadt, Germany), anti-PSMD9 (Cat. # PA5121663, ThermoFisher Scientific; Darmstadt, Germany), anti-HSP90 (Cat. # 610,419, BD Biosciences; Heidelberg, Germany), anti-β-actin (Cat. # MA1115, Boster Biologics; Hamburg, Germany), anti-p21 (Cat. # 2947, Cell Signaling Technology; Leiden, Netherlands), anti-p-CHK1 (Cat. # PA5-34,625, ThermoFisher Scientific; Darmstadt, Germany), anti-CHK1 (Cat. # 2360, Cell Signaling Technology; Leiden, Netherlands), anti-FERMT3 (Cat. # ab68040, Abcam; Amsterdam, Netherlands). Proteins were visualized by enhanced chemiluminescence using a commercially available kit SuperSignal™ West Femto Maximum Sensitivity Substrate (Cat. # 34,095; ThermoFisher Scientific; Darmstadt, Germany).

### Immunoprecipitation

Three days after adenoviral transduction, cells were washed once with PBS prior to collection in 700 µl of affinity purification lysis buffer containing Tris/HCl pH 7.5 (50 mmol/L), NaCl (150 mmol/L), NP-40 (1%), Na_4_P_2_O_7_ (10 mmol/L), NaF (20 mmol/L), orthovanadate (2 mmol/L), okadaic acid (10 nmol/L), β-glycerophosphate (50 mmol/L), phenylmethylsulfonyl fluoride (230 µmol/L) and an EDTA-free protease inhibitor mix (Applichem GmbH; Darmstadt, Germany GmbH, Darmstadt, Germany). Lysates were incubated for 45–60 min on an end-over-end rocker and gently vortexed until no cell clumps were visible. Protein concentration was determined using the Bradford method. Whole cell lysates (500 µg/sample) were incubated with 20 µl anti-FLAG affinity gel (A2220, Merck; Darmstadt, Germany) overnight on an end-over-end rocker. Samples were then centrifuged at 5000 rpm for 2 min and the supernatant was removed. FLAG immunoprecipitates were washed twice with affinity purification lysis buffer and centrifuged at 5000 rpm for 30 s at 4 °C. For proteomic analysis, FLAG immunoprecipitates were washed with a buffer containing 50 mmol/L Tris HCl (pH 7.5) and 150 mmol/L NaCl, heated in elution buffer containing Tris HCl pH 7.5 (50 mmol/L) and 2% SDS for 10 min at 95 °C and stored at −20 °C until further processing. For immunoblotting, FLAG immunoprecipitates were directly heated in elution buffer containing 2% SDS, 1% β-mercaptoethanol and 0.005% bromophenol blue in PBS for 10 min at 95 °C.

### Identification of the miP-FERMT3 interactome by LC–MS/MS

miP-FERMT3 interactomes were prepared for mass spectrometry-based proteomics using a modified single-pot, solid-phase-enhanced sample preparation (SP3) method [74], adapted for a 96-well plate format. As proteins were eluted in an SDS-containing buffer, the addition of a detergent for initial denaturation was not required. Sample preparation was initiated immediately with the thermal denaturation and reduction steps. Samples were incubated for 10 min at 90 °C with shaking at 1200 rpm in a ThermoMixer to ensure full denaturation, followed by the addition of the DTT reductant to begin the reduction step. The remainder of the SP3 protocol, including the subsequent alkylation, bead binding, extensive washing (to remove the SDS detergent), tryptic digestion and final peptide elution was performed as described on zenodo.org, record number: 17474920.

Peptide injection, separation and measurement on Bruker TimsTof Ultra as well as subsequent spectrum matching with DIA-NN [75] was performed as described on zenodo.org, record number: 17475274. The search was performed against the Human Uniprot.org database (reviewed Swiss-Prot entries; October 2022) complemented with the FLAG-miP sequence used (database is available in the repository, see section Data and materials availability).

Downstream data processing and statistical analysis were carried out by the Autonomics package developed in-house (10.18129/B9.bioc.autonomics:1.15.235). Proteins with a q-value of < 0.01 were included for further analysis. MaxLFQ log2maxlfq intensities were used for quantitation and missing values imputed. All intensities containing only 1 precursor (Np) per sample were exchanged by NA for that particular sample. Differential abundance of protein groups was evaluated by limma [76].

The full list of DIA-NN settings, DIA-NN output, R code for data processing and statistical analysis are uploaded along with the mass spectrometric raw data to the ProteomeXchange Consortium (see section Data and materials availability).

For the miP-FERMT3 interactome, proteins significantly (FDR ≤ 0.05) enriched in the FLAG-miP-FERMT3 pulldown compared to the EGFP (control) were analysed by STRING v.11.5 GO term enrichment analysis [77].

### Whole cell proteome analysis by LC-MS.^3^

Samples were prepared as previously described [78, 79] by lysis in SDS buffer, reduction with Tris(2-carboxyethyl)phosphine hydrochloride, alkylation with chloroacetamide, and purification by methanol/chloroform precipitation. Proteins were then resuspended in 8 mol/L urea and digested overnight at 37 °C with Lys-C (Wako Chemicals, Neuss, Germany) at 1:50 (w/w) and Trypsin (Promega, Walldorf, Germany) at 1:100 (w/w). Peptides were purified using C18 Sep-Pak cartridges (Waters, WAT054955) and labelled with TMTpro 18-plex reagents (ThermoFisher Scientific, A52045). An equal amount of each labelled sample was pooled and fractionated by high-pH reversed-phase micro-flow chromatography into 24 fractions. Fractionated peptides were analysed on an Orbitrap Ascend Tribrid Mass Spectrometer (ThermoFisher Scientific) coupled to a Vanquish Neo UHPLC (ThermoFisher Scientific) using a 35 cm C18 analytical column. Peptides were separated over a 90-min non-linear gradient (7–40% B) and acquired using a synchronous precursor selection (SPS) multi-notch MS3 method. MS1 scans (350–1400 m/z, 120,000 resolution) were followed by CID-MS2 (NCE 35%) in the ion trap using Top Speed selection (1.2 s, charge 2–6). SPS-MS3 was triggered by Real-time Search against the human SwissProt proteome supplemented with miP-FERMT3 (miP100) and miRFP670nano sequences; the 10 most intense MS2 ions were fragmented by HCD (NCE 55%) and detected in the Orbitrap (45,000 resolution, 100–200 m/z). Raw data were processed in Proteome Discoverer 2.4 (ThermoFisher Scientific) using a TMTpro SPS-MS3 workflow as described [80]. MS2 spectra were searched with SequestHT against the human SwissProt reference proteome combined with miP-FERMT3, miRFP670nano. TMTpro (+ 304.207 Da) at peptide N-termini and lysine residues, and carbamidomethylation (+ 57.021 Da) at cysteine residues were set as static modifications. Methionine oxidation (+ 15.995 Da) was set as a dynamic modification. Precursor and fragment mass tolerances were 7 ppm and 0.5 Da, respectively. FDR was controlled at < 1% (PSM and protein level) using Percolator. Reporter ions were quantified from MS3 spectra, filtered for signal-to-noise > 10 and SPS purity > 50%, and protein abundances were normalized to total peptide amount. Differential abundance analysis was performed using the NormalyzerDE package [81] version 1.9.1 with limma as the statistical framework. Proteins were considered significantly regulated at an FDR-adjusted *p*-value < 0.05 and an absolute log₂ fold change > 0.585 (corresponding to a 1.5-fold change).

### Functional assays

#### Cell proliferation and cell area

Twenty-four hours post adenoviral transduction or 48 h after transfection, cells were seeded at a density of 5,000 cells per well into a 96-well plate (day 0). After 3 h of attachment, cells were incubated and imaged for up to 5 days using Incucyte S3 live-cell imaging analysis system (Sartorius, Göttingen, Germany). Live cell count and average cell area were quantified by AI cell health analysis module (Segmentation sensitivity 0.5, Score threshold 0.2) within the Incucyte software.

#### Fluorescent Ubiquitination-based Cell Cycle Indicator (FUCCI) cell tracking

Cells expressing FUCCI were transduced with adenoviruses as described above. Similar to cell proliferation assay, cells were seeded and cultured at a density of 5000 cells per well into a 96-well plate (day 0). Images were acquired every hour using Incucyte S3 live-cell imaging and analysis system (Sartorius, Göttingen, Germany).

#### EdU incorporation

EdU assay was performed using Click-iT EdU Cell Proliferation Kit (Cat. # C10338; ThermoFisher Scientific; Darmstadt, Germany) according to the manufacturer’s instructions. Briefly, cells were incubated with 10 µmol/L EdU for 2 h. Cells were then fixed with 4% PFA for 15 min and permeabilized with 0.5% Triton X-100 for 20 min at room temperature. Following two washes with 3% bovine serum albumin in PBS, cells were incubated with Click-iT® reaction cocktail for 30 min in the dark. The total number of cells and EdU positive cells were quantified using the Incucyte S3 live-cell imaging and analysis system (Sartorius, Göttingen, Germany). Results were shown as percent of EdU +/total number of cells.

### RNA isolation, next-generation RNA sequencing and RT-qPCR

Four days after adenoviral transduction, cells were lysed for 45–60 min at 4ºC in a lysis buffer containing Tris–HCl pH7.5 (50 mmol/L, Applichem GmbH; Darmstadt, Germany), NaCl (150 mmol/L, Merck; Darmstadt, Germany), MgCl_2_ (10 mmol/L, Invitrogen, Schwerte, Germany), NP-40 (1%, Merck; Darmstadt, Germany) dissolved in UltraPure DNase/RNase-free Distilled water (Invitrogen, Darmstadt, Germany) and supplemented with NaPPi (10 mmol/L, Merck; Darmstadt, Germany), NaF (20 mmol/L, Applichem GmbH; Darmstadt, Germany), okadaic acid (10 nmol/L, LC laboratory, Massachusetts, USA), Na_3_VO_4_ (2 mmol/L, Sigma-Aldrich; Darmstadt, Germany), PIM, (12 µl/ml, Sigma-Aldrich; Darmstadt, Germany), Phenylmethylsulfonyl fluoride (4 µl/ml, Carl Roth, Karlsruhe, Germany), SUPERase In RNase Inhibitor (200U/ml, Invitrogen, Darmstadt, Germany), TURBO DNaseI (25U/ml, Invitrogen, Darmstadt, Germany). The cell lysate was passed 7–10 times through a 1 mL syringe fitted with a 26G needle to ensure complete disruption of cellular membranes. The suspension was then centrifuged at 13,000 rpm for 10 min at 4 °C. The resulting clear supernatant was carefully transferred to a new 1.5 mL microcentrifuge tube for RNA purification. Total RNA was purified using the miRNeasy Micro kit (QIAGEN; Hilden, Germany), according to the manufacturer’s instructions.

RNA and library preparation integrity were verified with LabChip Gx Touch (Perkin Elmer). RNA amounts were normalized and 500 ng/1 µg of total RNA was used as input for SMARTer Stranded Total RNA Sample Prep Kit HI Mammalian (Takara Bio). Sequencing was performed on the NextSeq2000 platform (Illumina) using P3 flowcell with 72 bp single-end setup. Trimmomatic version 0.39 was employed to trim reads after a quality drop below a mean of Q15 in a window of 5 nucleotides and keeping only filtered reads longer than 15 nucleotides [82]. Reads were aligned versus Ensembl human genome version hg38 (Ensembl release 109) with STAR 2.7.10a [83]. Alignments were filtered to remove: duplicates with Picard 3.0.0 (Picard: A set of tools (in Java) for working with next generation sequencing data in the BAM format), multi-mapping, ribosomal, or mitochondrial reads. Gene counts were established with featureCounts 2.0.4 by aggregating reads overlapping exons on the correct strand excluding those overlapping multiple genes [84]. The raw count matrix was normalized with DESeq2 version 1.36.0 [85]. Contrasts were created with DESeq2 based on the raw count matrix. Genes were classified as significantly differentially expressed at average count > 5, multiple testing adjusted *p*-value < 0.05, and −0.585 ≤ log2FC ≥ 0.585. The Ensemble annotation was enriched with UniProt data. Differentially expressed genes were analysed by STRING v.11.5 GO term enrichment analysis [77].

FERMT3 mRNA expression analysis in human mesenteric artery endothelial cells from 20-versus 80-year-old individuals was obtained from the publicly available dataset GSE214476 [37]. FERMT3 mRNA expression analysis of isolated cardiac endothelial cells from 2-versus 20-month-old mice was analysed as previously described [41].

Total RNA was extracted using TriReagent (Merck) according to the manufacturer’s protocol. For the generation of cDNA, total RNA was reverse transcribed using the SuperScriptIII (Life Technologies GmbH, Darmstadt, Germany) and random hexamer primers (Promega, Madison, USA). Messenger RNA levels were quantified using the cycle threshold (CT) values determined by SYBR green qPCR master mix (SensiFAST SYBR Lo-ROX Bioline, London, UK) in a MIC qPCR cycler (BMS, Upper Coomera, Australia). The follower primer pairs were used: Human 18S Forward: 5’-CTTTGGTCGCTCGCTCCTC-3’, and human 18S reverse: 5’-CTGACCGGGTTGGTTTTGAT-3’, human FERTM3 forward: 5’-CACCGAGCCACGCCCCCTGTGTCA-3’, human FERMT3 reverse: 5’-AAACTGACACAGGGGGCGTGGCTC-3’.

### Flow cytometry

Cells were seeded at a density of 100,000 cells in 6-cm dishes and harvested after 24 h (day 1) or 120 h (day 5). Cells were fixed with 4% PFA for 15 min at room temperature, then incubated with 20 μmol/L Hoechst 33,342 (B2261, Sigma-Aldrich; Darmstadt, Germany) for 45 min at 37 °C. After a single PBS wash, cells were resuspended in PBS and analysed by flow cytometry. At least 50,000 events per sample were recorded for quantification. Fluorescence was analysed using a BD LSR II/Fortessa flow cytometer (BD Biosciences; Heidelberg, Germany), and data were processed using FlowJo Vx software (TreeStar, UK). Daily instrument calibration was performed with Cytometer Setup and Tracking beads (BD Biosciences; Heidelberg, Germany).

### Senescence-associated β-galactosidase activity

Senescence-associated β-galactosidase activity was assessed using a Cellular Senescence assay kit (Cat. # KAA002, Merck; Darmstadt, Germany) according to the manufacturer’s instructions. In brief, cells were fixed with fixing solution (Part No. 2004755, 15 min at room temperature) 7 days post adenoviral transduction. After washing 3 times with PBS, cells were incubated with 150 µL of freshly prepared X-gal solution (Part No. 2004756, Part No. 2004754, Part No. 2004752) at 37 °C overnight. Cells were visualized in phase contrast mode using a Zeiss Axio Observer microscope (Zeiss; Oberkochen, Germany) and analysed with the ZEN software (Zeiss; Oberkochen, Germany). At least 200 cells per cell batch were imaged and quantified. Results were expressed as senescence-associated β-galactosidase positive cells/total number of cells.

### Proteasome activity assay

Proteasomal activity was measured using the Proteasome 20S Activity Assay Kit (MAK172, Merck; Darmstadt, Germany) according to the manufacturer’s instructions. Briefly, five days post-transduction or 96 h after transfection, cells were seeded in 96-well plates at a density of 40,000 cells per well and allowed to adhere for 3 h. Cells were imaged using the the Incucyte S3 live-cell imaging and analysis system (Sartorius; Göttingen, Germany) to determine the number of adherent cells per well as described above, followed by incubation with Proteasome Assay Loading Solution overnight at 37 °C. Fluorescence intensity was measured at λ_ex_ = 485 nm and λ_em_ = 535 nm, and background fluorescence (medium without cells) was subtracted from each sample fluorescence value. Proteasomal activity was normalized to the total number of cells.

### Telomere length

Telomere length was quantified using a monochrome multiplexing qPCR method as described previously [86]. Briefly, genomic DNA was isolated from endothelial cells five days post-transduction using the DNeasy Blood and Tissue Kit (Cat. No. 69504, Qiagen, Hilden, Germany). For PCR, 20 ng genomic DNA per reaction was used. Primer pairs used for telomere (T) amplification were telomere forward primer: 5′-ACACTAAGGTTTGGGTTTGGGTTTGGGTTTGGGTTAGTGT-3′ and telomere reverse primer: 5′–TGTTAGGTATCCCTATCCCTATCCCTATCCCTATCCCTAACA–3′. As single copy gene reference (S), human β-globin was amplified at 88 °C using the primers β-globin forward primer: 5′–CGGCGGCGGGCGGCCGGGGCTGGGCGGCTTCATCCACGTTCACCTTG–3′ and β-globin reverse primer: 5′–GCCCGGCCCGCCGCGCCCGTCCCGCCGGAGGAGAAGTCTGCCGTT–3′. All reactions were carried out in quadruplicate. Relative telomere length was calculated as the telomere-to-single-copy gene (T/S) ratio using the Pfaffl method [87] and normalized to the mean values of control samples.

### Collection of conditioned media and monocyte adhesion assay

Forty-eight hours after adenoviral transduction, the culture media was replaced with ECGM2 and incubated with the cells for 96 h. Conditioned media were subsequently used to treat naïve human endothelial cells for 24 h. THP-1 monocytes were stained using 200 nmol/L CellTracker Red CMTPX dye (Cat. # C34552, ThermoFisher Scientific; Darmstadt, Germany) for 15 min at 37 °C. After staining, cells were washed, counted, and resuspended in ECGM2 medium. Subsequently, 25,000 THP-1 cells were allowed to adhere to endothelial cells for 30 min. Non-adherent cells were removed by three thorough washes, and the number of adherent monocytes was quantified using the Incucyte S3 live-cell imaging and analysis system (Sartorius, Göttingen, Germany).

### Statistics

Results are presented as mean ± standard error of the mean. GraphPad Prism software (v. 10.1.2) was used to assess statistical significance. Differences between two groups were compared by two-tailed unpaired Student’s t-test. All experiments in which the effects of two variables were tested were analysed by two-way ANOVA followed by the Šídák's multiple comparisons test. Differences were considered statistically significant when *p*-value < 0.05. Only exact significant *p*-values are reported.

## Supplementary Information

Supplementary Material 1: Dataset 1.

Supplementary Material 2: Dataset 2.

Supplementary Material 3: Dataset 3.

Supplementary Material 4: Dataset 4.

Supplementary Material 5: Figure S1.

Supplementary Material 6: Figure S1. Subcellular localization of miP-FERMT3 in endothelial cells. Confocal images showing FLAG-miP-FERMT3 together with LAMP1 (lysosomes), EEA1 (early endosomes), Mitotracker (mitochondria), NOGO-B and calnexin (endoplasmic reticulum). Nuclei were stained with DAPI (blue). Similar results were obtained in 3-5 independent cell batches. Scale bar = 25 µm.

## Data Availability

Further information and requests for resources and reagents should be directed to and will be fulfilled by Dr. Mauro Siragusa (Siragusa@vrc.uni-frankfurt.de). Data that support the findings of this study are available as part of the manuscript. Raw data can be accessed at the following repositories: Mass spectrometry-based proteomics data: ProteomeXchange Consortium via the MassIVE and PRIDE [88] partner repositories (http://proteomecentral.proteomexchange.org) with the following identifiers: miP-FERMT3 interactome: PXD069811 (MassIVE ID: MSV000099566). Whole cell proteome: PXD077152. Bulk RNA-seq data are deposited in Gene Expression Omnibus (GEO) under the accession number GSE310464.http://proteomecentral.proteomexchange.org
