# Supplementary material for: A microprotein encoded by FERMT3 modulates endothelial cell protein catabolism and induces cell cycle arrest and senescence
Source: Cell Commun Signal. 2026 Jun 25;24:372. doi: 10.1186/s12964-026-03019-3 (PMC13295548; doi:10.1186/s12964-026-03019-3)

Full uncropped blots for Figure 2B

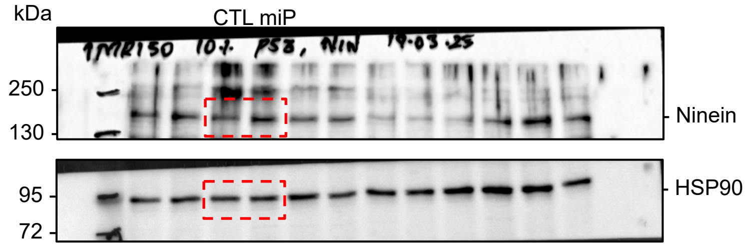

Full uncropped blots for Figure 2D

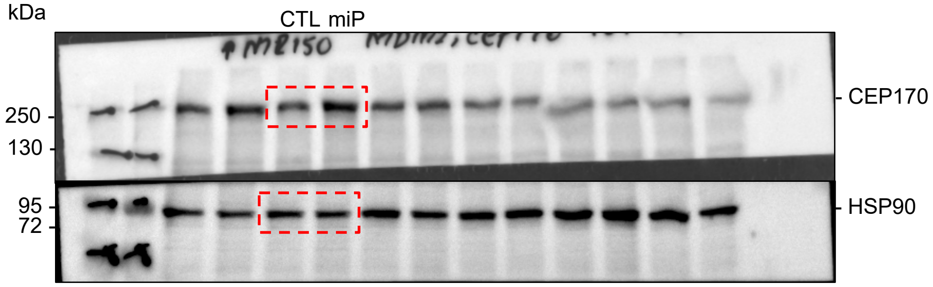

Full uncropped blots for Figure 2E

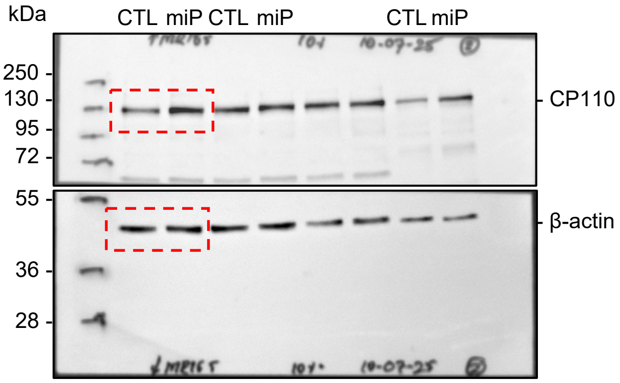

Full uncropped blots for Figure 2K

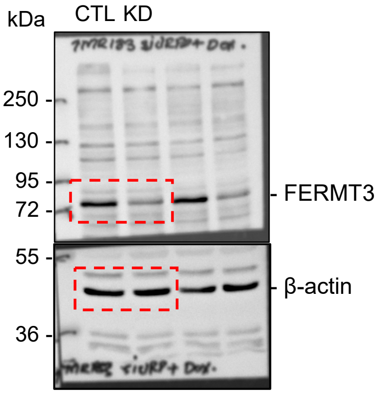

Full uncropped blots for Figure 4C

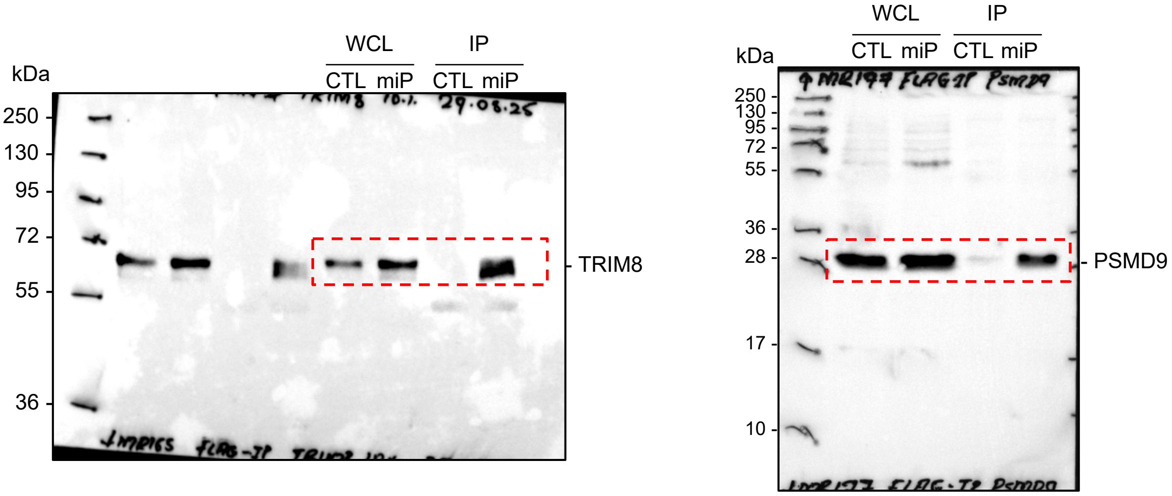

Full uncropped blots for Figure 4H

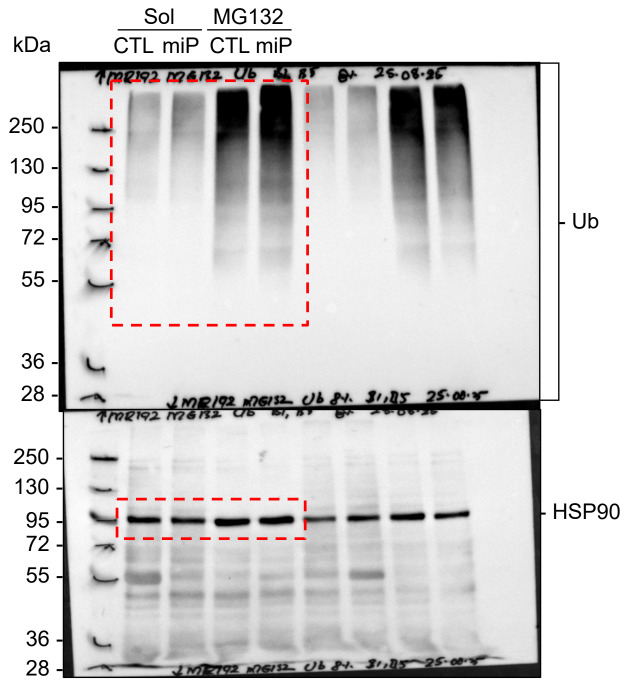

Full uncropped blots for Figure 5C

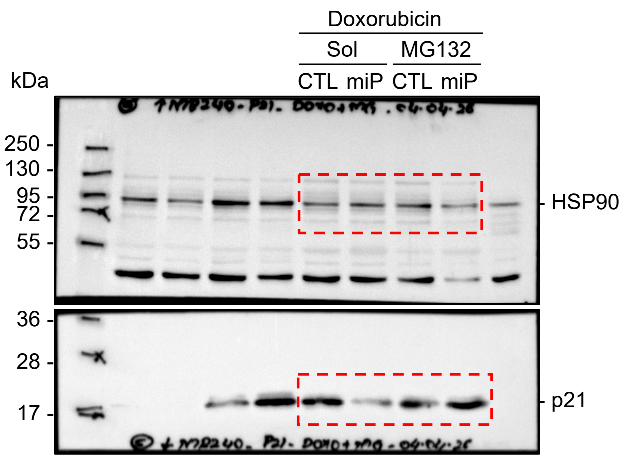

Full uncropped blots for Figure 5D

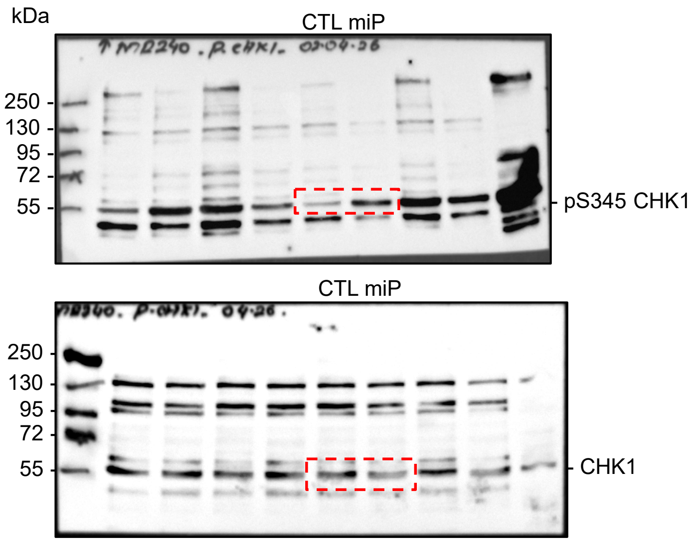

Supplement: Supplementary file 5 — Supplementary Material 5: Figure S1. [file 12964_2026_3019_MOESM5_ESM.pdf]
