# Supplementary figures and images for: A microprotein encoded by FERMT3 modulates endothelial cell protein catabolism and induces cell cycle arrest and senescence
Source: Cell Commun Signal. 2026 Jun 25;24:372. doi: 10.1186/s12964-026-03019-3 (PMC13295548; doi:10.1186/s12964-026-03019-3)

LAMP1

EEA1

Mitotracker

NOGO-B

Calnexin

FLAG-miP-FERMT3

Merge

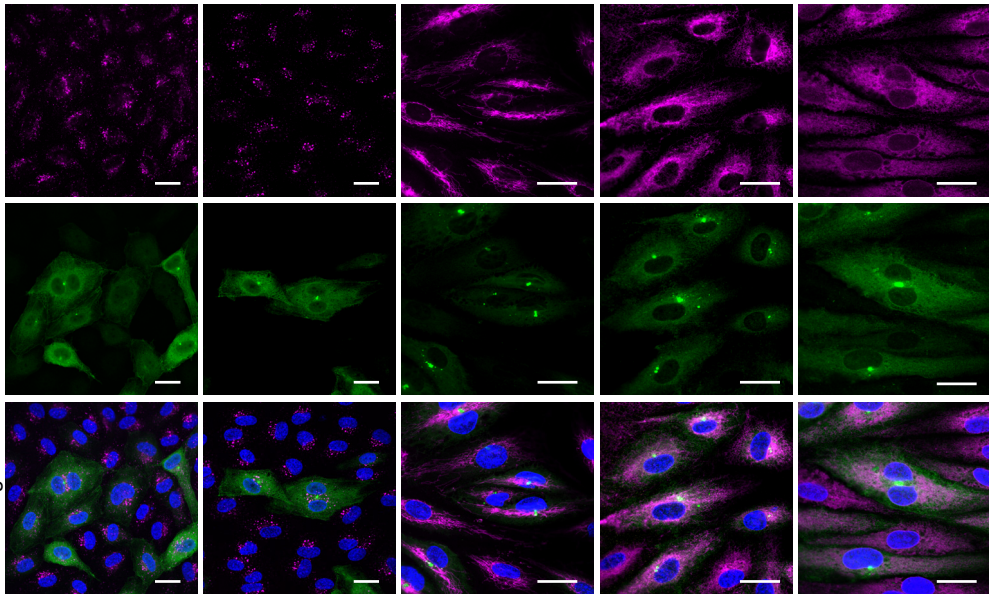

Supplement: Supplementary file 6 — Supplementary Material 6: Figure S1. Subcellular localization of miP-FERMT3 in endothelial cells. Confocal images showing FLAG-miP-FERMT3 together with LAMP1 (lysosomes), EEA1 (early endosomes), Mitotracker (mitochondria), NOGO-B and calnexin (endoplasmic reticulum). Nuclei were stained with DAPI (blue). Similar results were obtained in 3-5 independent cell batches. Scale bar = 25 µm. [file 12964_2026_3019_MOESM6_ESM.pdf]
